# Construction of novel radiomics nomogram model based on preoperative CT to predict lymphovascular tumor embolus and recurrence-free survival in early T1-2a stage lung adenocarcinomas

**DOI:** 10.1186/s12880-026-02240-3

**Published:** 2026-02-23

**Authors:** Junzhong Liu, Shiying Ju, Zhaofeng Zheng, Mingyuan Pang, Yujing Chu, Longjiang Fang, Linkun Li, Wenjuan Wang, Qi Wang

**Affiliations:** Department of Radiology, Weifang People’s Hospital, Shandong Second Medical University, Weifang, Shandong 261000 P.R. China

**Keywords:** Lymphovascular tumor embolus, Lung adenocarcinoma, Tomography, X-ray computed, Texture analysis, Radiomic

## Abstract

**Purpose:**

To construct a radiomics nomogram model predicting the status of lymphovascular tumor embolus (LTE) in patients with lung invasive adenocarcinoma (LAC).

**Materials and methods:**

This retrospective analysis enrolled 195 patients with pathologically-confirmed LAC, treated at Weifang People’s Hospital between January 2018 and April 2021, including 152 and 43 cases in the LTE and non-LTE groups, respectively. Regions of interest were manually delineated on preoperative CT images using 3D slicer. Subsequently, 850 radiomics features were extracted and subjected to feature reduction through least absolute shrinkage and selection operator regression. The effectiveness of the predictive model was evaluated using receiver operating characteristic curves, calibration, and decision curve analysis. The log-rank test was applied to data split into low-score and high-score groups to analyze early recurrence-free survival based on the optimal cutoff value established in the mixed model.

**Results:**

Five identified feature parameters were applied to establish a rad-score. Hybrid prediction model integrating smoking status and radiomics signature demonstrated better predictive efficacy than the radiomics models in the training cohort (area under the curve [AUC], 0.9210 vs. 0.8781) and validation cohort (AUC, 0.8807 vs. 0.8770), although without reaching statistical significance. The calibration curves of the nomogram illustrated the goodness-of-fit to predict LTE status in both cohorts. Kaplan-Meier survival curve analysis demonstrated a significant difference in recurrence-free survival rate between the low-score and high-score groups, as predicted based on the optimal cutoff value of the mixed model.

**Conclusion:**

CT radiomics-based model, which could serve as a potential biomarker, demonstrated strong predictive value for LTE status in LAC.

## Introduction

Lung cancer remains one of the most lethal malignancies worldwide, with the highest morbidity and mortality rates [1]. Among non-small cell lung cancers (NSCLCs), lung adenocarcinoma (LAC) is the most common histological subtype. Over the past decade, the proportion of patients diagnosed with clinical stage I lung cancer has increased, largely due to the early diagnostic application of low-dose computed tomography. Sublobectomy is considered the most effective treatment option for patients with stage I NSCLC. However, outcomes remain inadequate due to tumor recurrence following surgical resection. Patients with stage I NSCLC show a recurrence rate of 33%, with a five-year survival rate of 51% [2]. Over 50% of patients undergoing curative treatment experience recurrence within five years, and the overall survival rates at five years continue to be suboptimal [3, 4]. Based on this, it is imperative to investigate factors that could be used to predict metastasis and recurrence following resection in clinical early-stage lung adenocarcinoma, as this knowledge will be vital for patient stratification and management.

Lymphovascular tumor embolus (LTE) refers to the formation of cancer thrombus after malignant cells invade arteries, veins, or lymphatic vessels, and is an important risk factor for predicting disease recurrence and metastasis [4–8]. Tumor metastasis caused by LTE leads to recurrence and death in patients. Research has indicated that LTE is an important mechanism underlying tumor metastasis, involving the diffusion of cancer cells via the circulatory system [9]. Patients classified as stage IA or IB with microvascular invasion (MVI) exhibited outcomes similar to those seen in patients with stage IB or IIA NSCLC [10, 11]. The National Comprehensive Cancer Network (NCCN) guidelines for NSCLC designate MVI as a “high-risk factor,” recommending that patients with stage IB NSCLC who present with this condition should undergo systemic therapy, including adjuvant chemotherapy [10, 12]. Accordingly, more in-depth research into the occurrence and development of LTE could help to prevent solid tumor metastasis and improve patient survival rates. LTE is a histologically-defined condition that can only be confirmed after surgery through the examination of a collected specimen. Limited imaging characteristics have been identified that can help to anticipate an ultimate diagnosis of LTE in cases of lung cancer.

Although conventional contrast-enhanced CT is diagnostically capable of detecting macrovascular thrombi in NSCLC, it is unable to detect MVI, particularly LTE [13, 14]. CT radiomics, an innovative imaging analysis approach employing data-mining algorithms and statistical tools to extract predictive insights from high-throughput imaging features, can not only quantitatively analyze the uneven tumor texture caused by lymphatic vessel growth, but can also reveal tumor heterogeneous phenotypic characteristics [5, 10, 15, 16]. This technique has shown notable clinical advantages in MVI-positive hepatocellular carcinoma by developing radiomics nomograms that integrate refined CT features with clinical variables, demonstrating strong potential for precision assessment [16–19]. There are relatively few studies on MIV in early-stage lung cancer based on radiomics [5, 10, 11, 13, 14]. Furthermore, there are still no studies investigating the prediction of LTE as a subgroup of MVI, or its relationship with recurrence-free survival (RFS).

In this context, the present study aimed to examine whether a nomogram that combined clinical and CT radiomics features could effectively forecast LTE status and RFS in patients diagnosed with early-stage NSCLC.

## Materials and methods

### Ethics approval

As this was a retrospective study, the Weifang People’s Hospital Ethics Committee granted exemption from obtaining informed consent (Ethics Review No. KYLL20250521-1) while approving all study procedures in compliance with the Declaration of Helsinki.

### Study design and patients

**Fig. 1:**
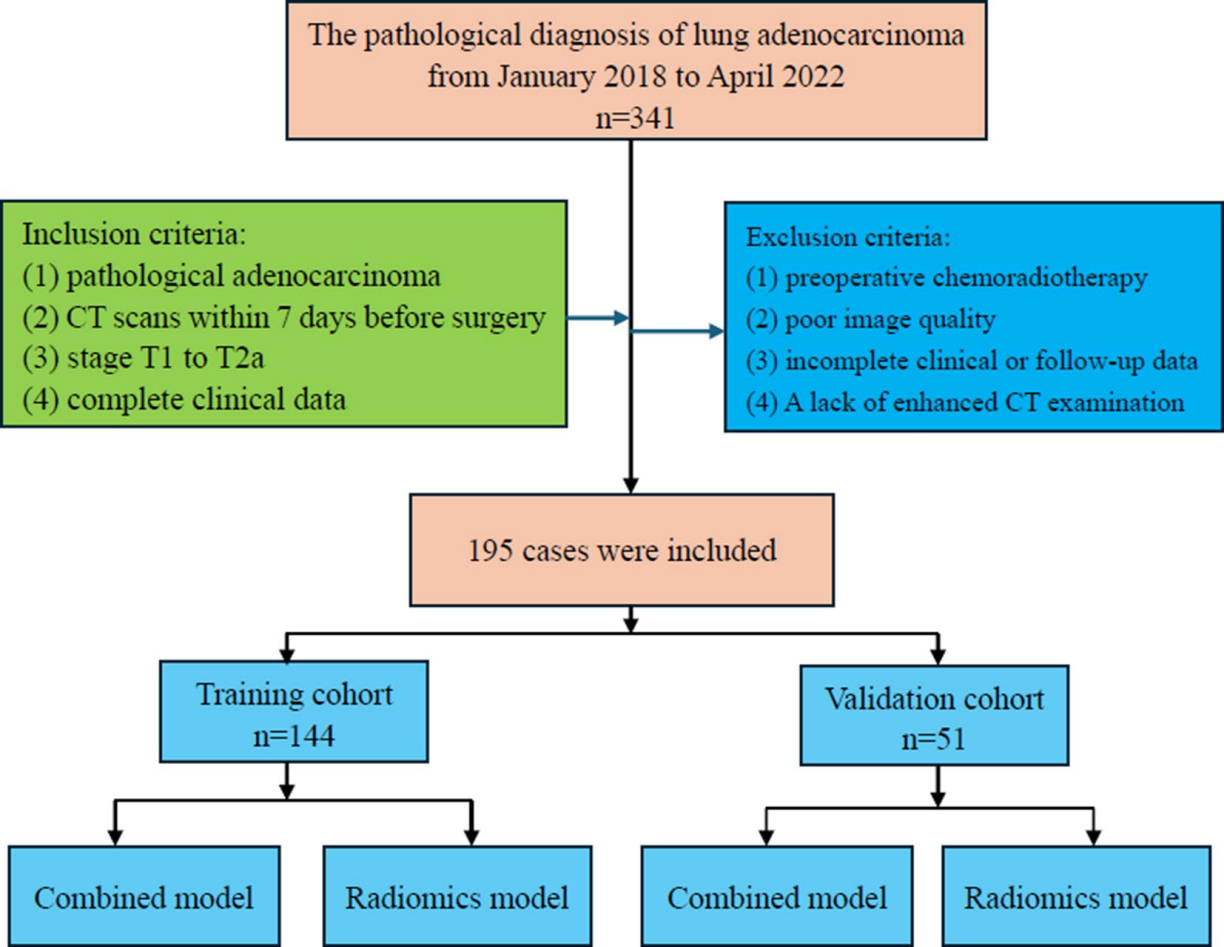
Flow chart illustrating the patient selection and exclusion criteria

The pathological diagnosis of LAC in lung cancer surgery patients was established through a review of medical records at the Weifang People’s Hospital of Shandong Second Medical University, from January 2018 to April 2022. The inclusion criteria were: (1) patients with a confirmed pathological diagnosis of solid LAC; (2) patients who underwent chest CT scans at our hospital within 7 days prior to surgery; (3) a lung cancer stage of T1 to T2a; and (4) complete clinical data. The exclusion criteria included: (1) patients who received chemotherapy or radiotherapy before surgery; (2) poor image quality that may interfere with analysis; (3) incomplete clinical data or follow-up information; (4) A lack of enhanced CT examination. Ultimately, a total of 195 cases were included, comprising 98 men and 97 women, among whom 43 were diagnosed with LTE and 152 did not have LTE. Figure 1 presents a detailed overview of the inclusion and exclusion criteria and the associated processes.

The status of LTE in surgical resection specimens was characterized by the presence of tumor thrombus within the lymphatic, arterial, or venous vessels of malignant tissue that could be identified under a microscope.

### Images acquisition and feature extraction

All patients underwent CT image acquisition using a 64-slice spiral CT scanner (GE Healthcare, USA) with the following parameters: tube voltage set at 120 kV, tube current ranging from 10 to 400 mA (employing automatic tube current modulation technology), a pitch of 1.375:1, a matrix size of 512 × 512, a scanning layer thickness of 5 mm, and an image reconstruction thickness of 1.25 mm. All patients underwent the breath-holding examination while in a supine position, with the scanning area encompassing both lungs. The subsequent CT characteristics were assessed in venous phase using lung window settings (width: 1500 HU; level: − 700 HU) and the mediastinal window (width: 350 HU; level: 40 HU).

Two radiologists, with 7 and 16 years of experience in thoracic imaging, respectively, blinded to the patients’ clinical information, identified the regions of interest in enhanced CT images using ITK-SNAP software from the picture archiving and communication system.

**Fig. 2:**
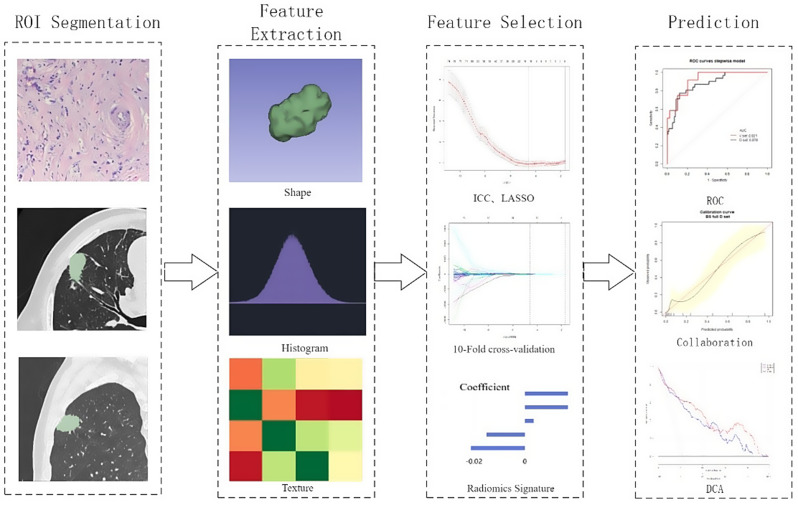
A standard workflow for radiomics research aimed at predicting lymphovascular tumor embolus in early T1-2a stage lung adenocarcinomas

Pyradiomics (version3.1.0) implemented in Python (version 3.7) was applied to extract radiomic features from CT figures, including first-order features, shape features (2D and 3D), and gray level features, such as the gray level co-occurrence matrix, gray level size zone matrix, gray level run length matrix, neighboring gray tone difference matrix, and gray level dependence matrix, as well as wavelet features. No additional preprocessing was performed since all CT images were obtained from a single institution using the same imaging protocol. The workflow of the study is presented in Fig. 2.

### Features selection and radiomics model construction

Radiomics features with intraclass and interclass correlation coefficients exceeding 0.75, following z-score normalization, were deemed identical and preserved for the training cohort. Least absolute shrinkage and selection operator (LASSO) analysis, combined with tenfold cross-validation, was conducted to identify the most predictive features and to reduce the dimensionality of the radiomics data. Subsequently, the radiomics score (RS) for each patient was calculated based on the features selected to construct radiomics model classifier by logistic regression. We further developed a model to predict LTE status by integrating various patient data, referred to as the hybrid model.

### Model construction and validation

Clinically significant factors identified in the univariable analysis, along with RS, were incorporated into the nomogram to predict the patient’s LTE status. Receiver Operating Characteristic curves (ROC), calibration curves, and decision curve analysis were applied to assess the performance, clinical benefits, and utility of the nomogram respectively.

### Follow-up and survival

Patients underwent CT evaluations every six months for the first two years following thoracic surgery, followed by annual assessments for up to five years. In total, 195 individuals were monitored for a minimum of five years. The research termination was designated as RFS, which refers to the interval between surgery and the occurrence of recurrence, metastasis, death, or the terminal reexamination. Significant variables identified in univariate analysis were incorporated into multivariate Cox regression to determine independent predictors of RFS. Survival curves were generated using the Kaplan-Meier method, with differences between high-score and low-score groups assessed using log-rank tests.

### Statistical analysis

All analyses were performed using Empower (R) (http://www.empowerstats.com, X&Y solutions, Inc., Boston, MA, version 5.2) and R (https://www.r-project.org, version 4.42). Univariate and multivariate logistic analyses were applied to identify independent predictors of LTE from clinical factors. Statistical tests with *p* values < 0.05 were considered significant. In addition, we calculated the sensitivity, specificity, accuracy, positive predictive value (PPV), positive likelihood ratio (PPV), negative likelihood ratio, diagnosis odds ratio, positive predictive value (PPA), and negative predictive value (NPV) of the models.

## Results

### Patient characteristics

**Fig. 3:**
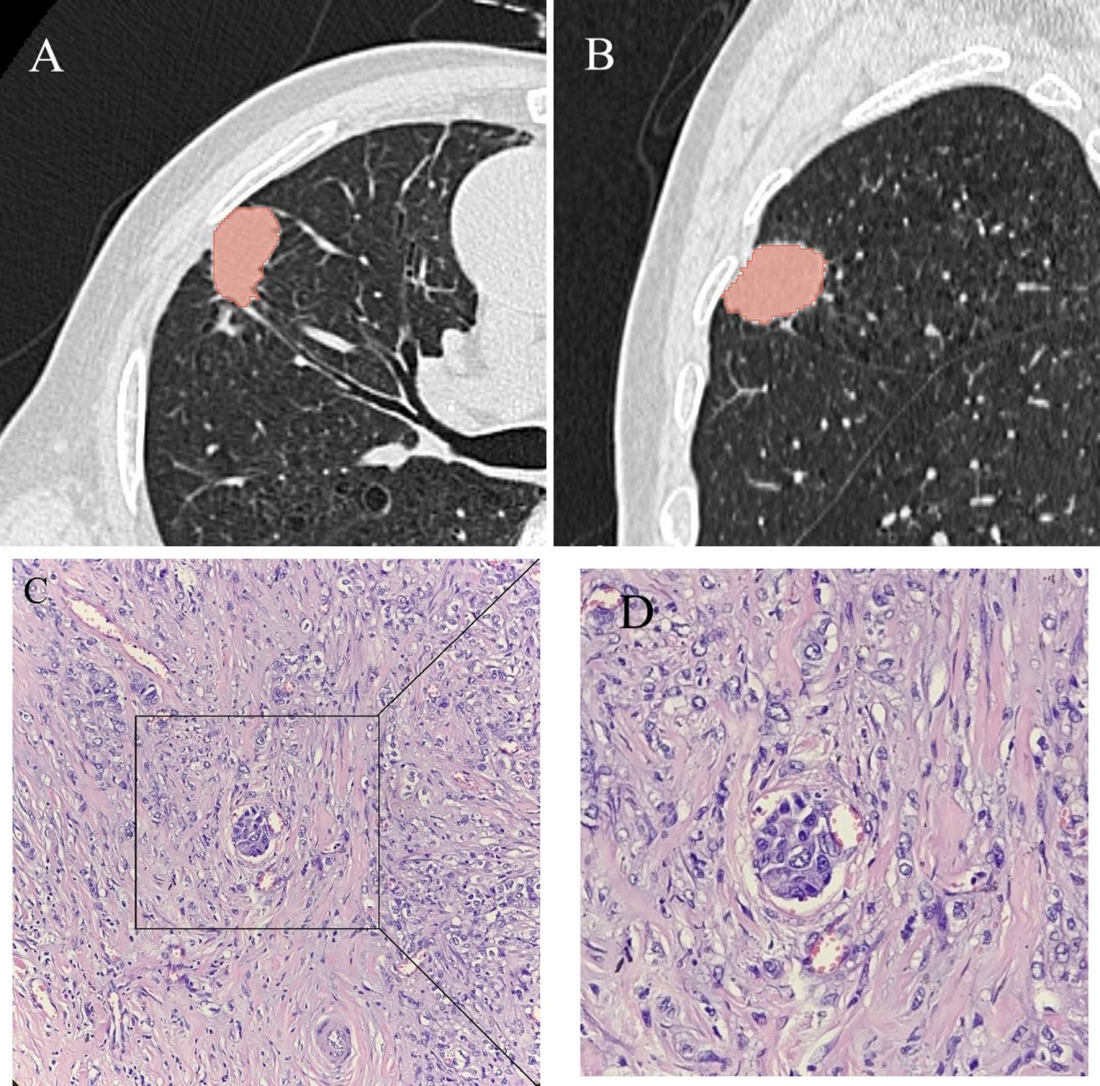
Representative CT and histopathological images illustrate a 65-year-old lung carcinoma patient whose lymphovascular Tumor Embolus (LTE) status was accurately predicted by the hybrid model. (**A**,** B**) A mass in the upper lobe of the right lung is segmented using 3Dslicer software. (**C**,** D**) Postoperative pathology showed adenocarcinoma with LTE (hematoxylin and eosin-stained sections)

Table 1 presents the comparisons of LTE status, demographic characteristics, and clinical features between the non-LTE and LTE cohorts. No significant differences were observed in age, sex, tumor location, or biochemical indicators between the non-LTE and LTE cohorts (*p*>0.05). A total of 195 patients (98 males and 97 females; mean age: 60.95 ± 9.06 years, age range: 28–83 years) were enrolled in this study. The training cohort comprised patients with 113 non-LTE and 31 LTE (*n* = 144), while the validation cohort comprised patients with 39 non-LTE and 12 LTE (*n* = 51). Univariate analysis revealed that smoking (*p* = 0.004; odds ratio [OR] = 2.89, 95% confidence interval [CI] = 1.05–7.99) and radiomics features (*p* = 0.000; OR = 3.32, 95% CI = 2.09–5.27) were independent indicators of LTE (Table 2), and these were subsequently used to construct the predictive model. Smoking status and radiomics signature were closely associated with the occurrence of LTE in lung cancer, as shown by the results of the univariate analysis in Table 2. An example of a representative case is illustrated in Fig. 3.

**Table 1:** Characteristics of patients in the non-LTE and LTE cohorts

Variable	Non-LTE Cohort	LTE Cohort	*P* value
Age (year)	60.55 ± 9.07	62.40 ± 8.98	0.238
Sex, n (%)			0.833
male	77 (50.66%)	21 (48.84%)	
female	75 (49.34%)	22 (51.16%)	
Smoke, n (%)			0.001
no	101 (66.45%)	17 (39.53%)	
yes	51 (33.55%)	26 (60.47%)	
Location, n (%)			0.387
Left upper lobe	34 (22.37%)	14 (32.56%)	
Left lower lobe	23 (15.13%)	8 (18.60%)	
Right upper lobe	56 (36.84%)	15 (34.88%)	
Right middle lobe	13 (8.55%)	1 (2.33%)	
Right lower lobe	26 (17.11%)	5 (11.63%)	
CEA (0-10ug/l)	2.46 ± 2.87	3.05 ± 2.42	0.221
NSE (0-20ng/ml)	12.52 ± 3.44	13.63 ± 4.64	0.087
CA125 (0-35U/ml)	4.61 ± 11.26	2.66 ± 4.64	0.270
Cyfra21-1(0-3.3ng/ml)	2.50 ± 2.46	2.57 ± 1.73	0.860
CA19-9 (0-27U/ml)	9.12 ± 8.20	7.90 ± 5.91	0.363
Surgical approach, n (%)			0.932
Sublobar resection	93 (61.18%)	26 (60.47%)	
Lobectomy resection	59 (38.82%)	17 (39.53%)	
Histologic subtype, n (%)			0.081
Lepidic pattern	20 (13.16%)	7 (16.28%)	
Acinar pattern	118 (77.63%)	28 (65.12%)	
Papillary pattern	9 (5.92%)	2 (4.65%)	
Micropapillary pattern	3 (1.97%)	3 (6.98%)	
Solid pattern	2 (1.32%)	3 (6.98%)	
Pleural invasion			0.052
no	143 (94.08%)	36 (83.72%)	
yes	9 (5.92%)	7 (16.28%)	

LTE, lymphovascular tumor embolism; CEA, carcino-embryonic antigen; NSE, neuron-specific enolase; CA125, carbohydrate antigen 125; Cyfra21-1, cytokeratin 221-1 fragments; CA19-9, Glucoprotein antigen 19 − 9

**Table 2:** Risk factors for lymphatic tumor embolism in stage IB lung adenocarcinoma

	β	Odds ratio (95% CI)	*P-value*
Smoke status	1.0629	2.8949(1.0476–7.999)	0.0040
Radiomics signature	1.2008	3.3226(2.0940–5.2721)	0.0001

### Development and assessment of predictive model

**Fig. 4:**
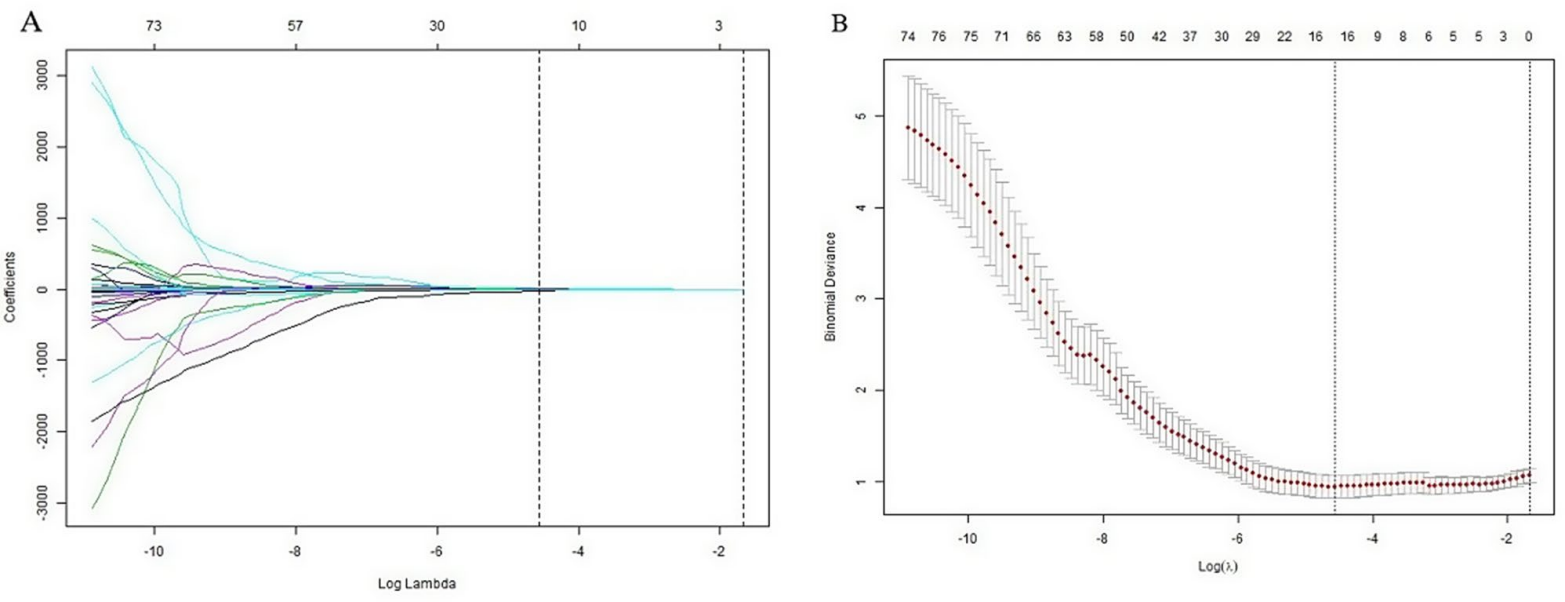
Radiomics feature selection using the least absolute shrinkage and selection operator (LASSO) binary logistic regression model. (**A**) Tuning parameter (lambda) selection in the LASSO model using 10-fold cross-validation. Dotted vertical lines represent the optimal values. The value of 0.0105, with log (λ) = − 4.5594 was chosen. (**B**) Graph of changes in the coefficient with the λ parameter. The dotted line corresponds to the best k value where the 5 features were retained

**Fig. 5:**
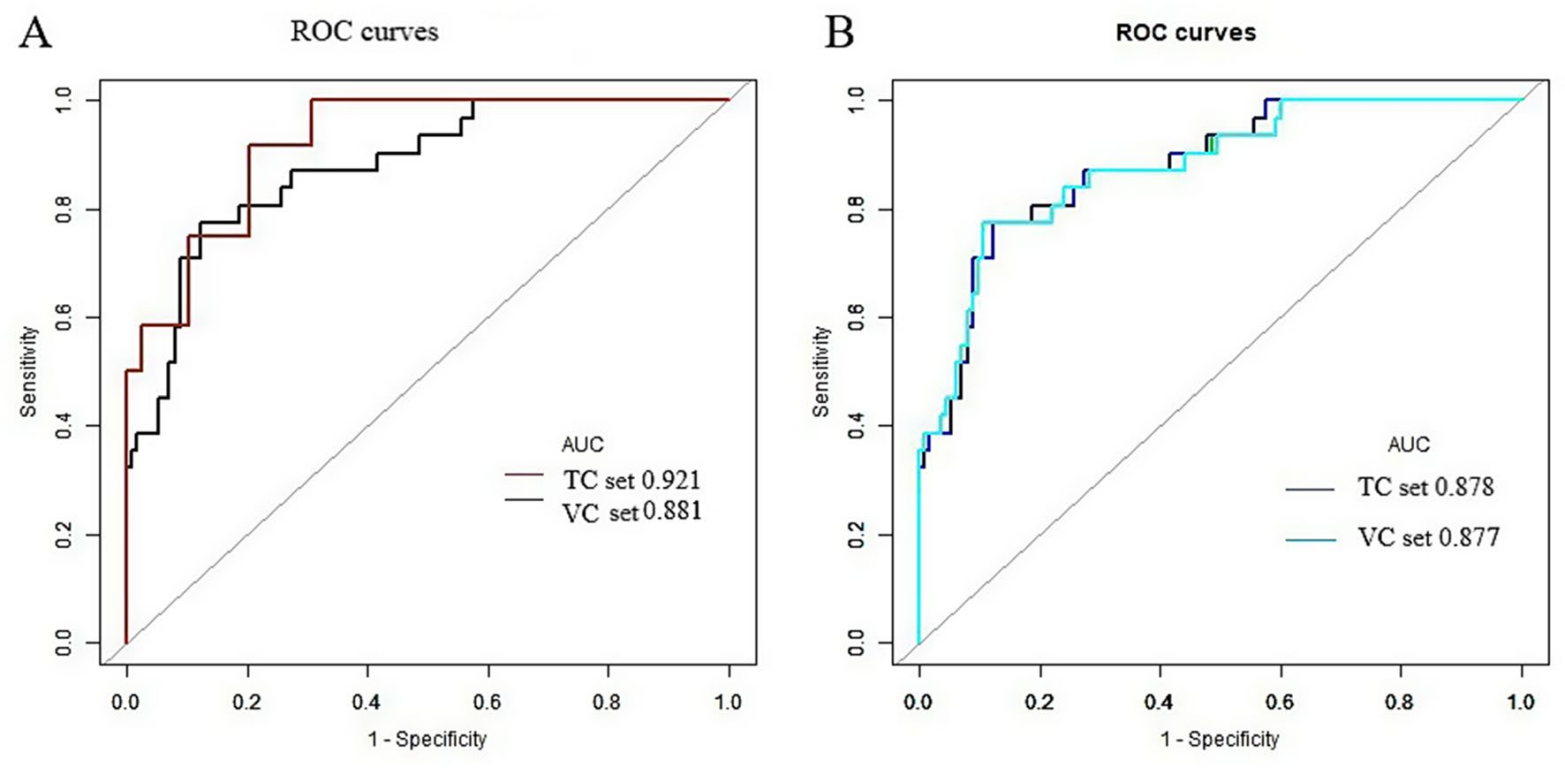
ROC and calibration curves of the training (**A**) and validation cohorts (**B**). The AUCs of the combined and the radiomics models in the training and validation cohorts were 0.921 and 0.881, and 0.878 and 0.877, respectively

**Fig. 6:**
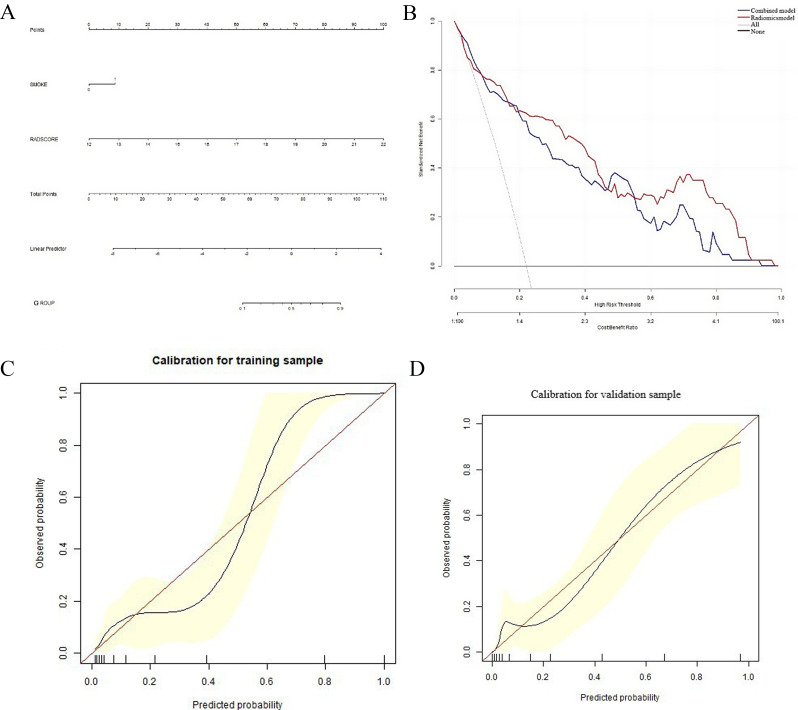
Nomogram of the lymphovascular tumor embolus(LTE)prediction model, Calibration and Decision curve analysis (DCA) of the combined and the radiomics nomogram. (**A)** The nomogram integrating radiomics and clinical factors to predict LTE. (**B**) DCA for the combined and radiomics models. (**C**,** D)** The calibration curves of the nomogram illustrated the goodness-of-fit to predict LTE status in the training and validation cohorts

In total, 5 out of 850 radiomics features were retained for model construction. The turning parameter (lambda) selection in the LASSO model used 10-fold cross-validation. A value of 0.0105, with log (λ), − 4.5594 was chosen. RS = 0.08199676*LongRunHighGrayLevelEmphasis + 0.0292689428*Autocorrelation + 0.00343896*Idn -0.01507002*DependenceNonUniformityNormalized -0.021191163 *Skewness + 0.21794872. Figure 4 presents the selection of radiomics features using LASSO in a binary logistic regression model. The AUC values of radiomics and combined models were 0.8807(CI, 0.8131–0.9482) and 0.925(CI, 0.858–1.0) in the training cohort (TC), and 0.8770(CI, 0. 8078–0.9461) and 0.8781(CI, 0.8114–0.9449) in the validation cohort (VC), respectively (Table 3). There was no significant difference in the AUC between the two models in paired comparisons (*p* > 0.05); however, the mixed model demonstrated superior performance compared to the radiomics model (Fig. 5). The hybrid prediction model integrating smoking status and radiomics signature is presented in the form of a nomogram (Fig. 6). The calibration curves of the nomogram illustrated the goodness-of-fit to predict LTE status in the TC and VC (Fig. 6).

**Table 3:** The diagnostic efficacy of the CT radiomics model and the combined model in the training set and validation cohort to predict lymphatic tumor embolism

	Training cohort(*n* = 144)		Validation cohort(*n* = 51)	
Model	CM	RM	CM	RM
AUC	0.9210	0.8807	0.8781	0.8770
Specificity	0.8974	0.8850	0.8938	0.8761
Sensitivity	0.8333	0.7742	0.7742	0.7742
Accuracy	0.8824	0.8611	0.8681	0.8542
PLR	8.1250	6.7295	7.2903	6.2488
NLR	0.1857	0.2552	0.2526	0.2577
DOR	43.7500	26.3736	28.8571	24.2449
PPV	0.7143	0.6486	0.6667	0.6316
NPV	0.9659	0.9346	0.9352	0.9340

CM, Combined model; RM, Radiomics model; AUC, area under the curve; CI, confidence interval; PLR, positive likelihood ratio; NLR, negative likelihood ratio; DOR, diagnosis odds ratio; PPV, positive predictive value; NPV, negative predictive value

### Survival prediction

**Fig. 7:**
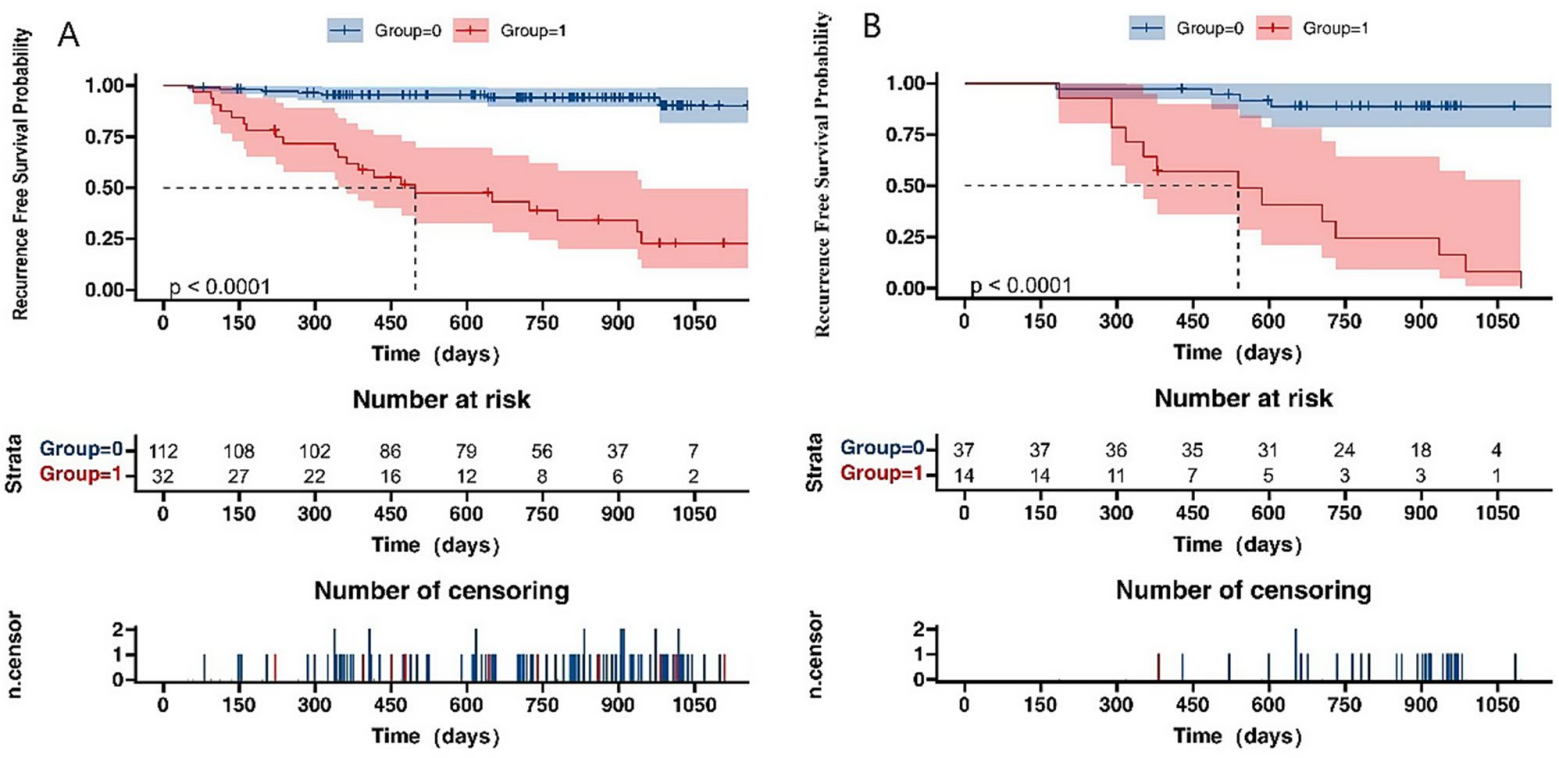
Kaplan-Meier curves of early recurrence-free survival (RFS) in the outcome set. (**A**,** B**) The 1-, 2-and 3-year recurrence-free survival were estimated using scores derived from combined model in the training and validation cohorts. A significant divergence in RFS rates was observed between the low-score and high-score groups via Kaplan-Meier survival analysis (*p* < 0.05), as anticipated by the optimal cutoff value established in the mixed model

After applying the inclusion criteria, 195 patients were included in the RFS analysis. The median RFS for these patients was 24 months (range, 2–40 months). The rates of RFS at 1-, 2-and 3-years postoperatively were 83.9%, 80.9%, and 61.1%, respectively. Kaplan-Meier survival curve analysis demonstrated a significant difference in the RFS rate between the low-score group and high-score group, as predicted based on the optimal cutoff value of the mixed model (*p*<0.05). The relevant survival curves are illustrated in Fig. 7.

## Discussion

LTE is a critical factor in the onset of tumor recurrence and metastasis. Consequently, accurately predicting LTE status preoperatively in patients with LAC is essential for establishing optimal treatment strategies. This study aimed to develop a predictive model for LTE status and RFS in early T1-2a stage LAC by integrating machine learning-derived radiomic signatures from preoperative CT scans with smoking status. The resulting model demonstrated high accuracy in forecasting LTE status and RFS for stage I LAC, demonstrating a favorable AUC as well as excellent sensitivity, specificity, NPV, PPV in the development and external validation cohorts.

MVI, which is an essential risk factor for predicting recurrence and metastasis, is a pathological result that can only be confirmed in tissue specimens obtained after surgical resection, making the preoperative prediction of lymphovascular invasion (LVI) particularly challenging with traditional imaging methods. Prior research has further shown that radiomics features could act as promising biomarkers for predicting LVI in different types of tumors [10, 16, 18, 19]. Previously, radiologists attempted to predict MVI based on clinical and routine CT features, resulting in unstable specificity, sensitivity, and accuracy due to the subjective influence of doctors [14]. In the past few years, there has been an increasing amount of research into the use of radiomics to predict MVI. For example, Xia et al. [19] used a CT-based radiomics model to predict MVI in hepatocellular carcinoma with the total radiomics model achieving an AUC of 0.76 in the internal test set and an AUC of 0.72 in the external test set. Another study on predicting MVI in intrahepatic cholangiocarcinoma based on multi sequence MR radiomics also achieved excellent predictive efficiency in the TC (AUC = 0.995) and VC (AUC = 0.867) [15]. Many scholars have similarly constructed different radiomics models to predict the vascular tumor thrombus status of liver cancer, including using CT, MRI, or PET/CT for MVI prediction [18–22]. Together, the results of these studies prove the feasibility of using radiomics in predicting vascular cancer thrombus.

This study revealed several radiomics features and established smoking status as an independent risk factor for LTE in early-stage NSCLC. In a previous study, Deng et al. [10] found that radiomics features consisting of one shape feature and four textural features performed well in predicting MVI in stage I NSCLC. Notably, their model configuration aligns closely with our own findings, featuring identical feature selection (Autocorrelation), which suggests that this feature may serve as a critical biomarker for MVI and holds significant clinical implications. However, like most studies, this study focused solely on MVI and did not address the subgroup of LTE formation. In this study, a statistically significant divergence in RFS was observed between the LTE-negative and LTE-positive cohorts through Kaplan-Meier survival analysis (*p* < 0.05). ln addition, smoke exposure demonstrated significant covariation with LTE phenomena in LAC cohorts, as evidenced by previous report [14]. Smoke exposure remodels the tumor vasculature architecture through HIF-1α/VEGF axis dysregulation, generating leaky vessels that facilitate tumor emboli entrapment [23, 24].

Numerous studies have found that the combination of intratumoral and peritumoral radiomics demonstrates a strong predictive capacity for MVI [14, 21, 25–29]. Peritumoral radiomic features may provide unique and valuable information reflecting tumor aggressiveness. Compared to radiomic features derived from a single region, integrating features from ‌intratumoral and peritumoral regions‌ could offer a more comprehensive understanding of the tumor’s pathophysiological characteristics. Nevertheless, Jiang et al. [30] found that intratumoral radiomics features extracted from digital mammography, digital breast tomosynthesis, dynamic contrast-enhanced, and diffusion-weighted magnetic resonance imaging (DWI-MRI) data outperformed peritumoral radiomics features in predicting breast cancer Ki-67 status. The rationale for not developing combined intratumoral-peritumoral models in the present study is based on the following considerations: firstly, while ‌peritumoral regions‌ play a critical role in evaluating tumor aggressiveness, ‌intratumoral regions‌ are more representative of tumor proliferation and heterogeneity [31]; secondly, the ‌optimal boundary definition for peritumoral regions‌ remains unstandardized across studies, introducing variability in feature extraction and model generalizability. The model developed in the present study can provide highly detailed biological information on intratumoral regions, and may also help to streamline clinical application workflows.

Despite the abovementioned strengths of this study, several methodological constraints should be acknowledged. First, this study followed a retrospective design, and focused solely on stage T1-2a LAC cases, which may lead to selection bias due to the exclusion of patients with advanced-stage malignancies. Second, although internal validation demonstrated model reliability, the absence of external validation using independent cohorts (e.g., TCIA datasets), combined with the limited sample size of the study cohort, may have potentially compromised predictive accuracy. Future prospective multicenter studies with larger cohorts are required to validate these findings and improve clinical applicability.

In conclusion, our radiomics-hybrid model developed from preoperative CT data could effectively predict LTE status and RFS in early T1-2a stage lung adenocarcinomas, which will aid in timely adjustments to treatment plans and facilitate prognostic risk stratification.

## Data Availability

All the data used in this work are available from the corresponding author upon reasonable request.

## Abbreviations

LTE: Lymphovascular tumor embolus
LAC: Lung invasive adenocarcinoma
AUC: Area under the curve
NSCLCs: Non-small cell lung cancers
MVI: Microvascular invasion
NCCN: National Comprehensive Cancer Network
RFS: Recurrence-free survival
LASSO: Least absolute shrinkage and selection operator
RS: Radiomics score
ROC: Receiver Operating Characteristic curves
PPV: Positive predictive value
PPA: Positive predictive value
NPV: Negative predictive value
OR: Odds ratio
CEA: Carcino-embryonic antigen
NSE: Neuron-specific enolase
CA125: Carbohydrate antigen 125
Cyfra21-1: Cytokeratin 21 − 1 fragments
CA19-9: Glucoprotein antigen 19 − 9
TC: Training cohort
VC: Validation cohort
CI: Confidence interval
PLR: Positive likelihood ratio
NLR: Negative likelihood ratio
DOR: Diagnosis odds ratio
DCA: Decision curve analysis
